# Oviductal fluid counterbalances the negative effect of high temperature on sperm in an ectotherm model

**DOI:** 10.1242/bio.058593

**Published:** 2021-04-15

**Authors:** N. Rossi, G. Lopez Juri, M. Chiaraviglio, G. Cardozo

**Affiliations:** 1 Universidad Nacional de Córdoba, Facultad de Ciencias Exactas Físicas y Naturales. Laboratorio de Biología del Comportamiento, X5000 Córdoba, Argentina; 2 Consejo Nacional de Investigaciones Científicas y Técnicas (CONICET), Instituto de Diversidad y Ecología Animal (IDEA), X5000 Córdoba, Argentina

**Keywords:** Sperm dynamic, Sperm motility, Temperature, Oviductal fluid, Global warming

## Abstract

Global warming is affecting biodiversity; however, the extent to which animal reproductive processes respond to predicted temperature increments remains largely unexplored. The thermal environment has a pronounced impact on metabolic rates of ectotherms; therefore, an interesting question to assess is whether temperature increase might affect specific reproductive mechanisms like sperm performance in ectotherms. Moreover, in many species, oviductal fluid (OF) is known to regulate and maintain sperm quality; however, the role of OF in relation to the effects of high temperature on sperm remains unclear. Our aim was to experimentally test the effect of increased temperature on sperm velocity, swimming path and percentage of motility in neutral conditions at ejaculation (without OF) and in female's reproductive tract fluid (with OF), in a social ectotherm lizard model, *Tropidurus spinulosus*, which has specific thermal requirements for reproduction. Our results suggest that a rising temperature associated with global warming (+4°C) affects negatively sperm dynamics and survival. However, OF ameliorated the harmful effects of high temperature. This is an important point, as this study is the first to have tested the role of OF in preserving sperm from a warmer pre-fertilization environment. These results contribute to our understanding of how thermal environment changes might affect post-copulatory reproductive mechanisms.

This article has an associated First Person interview with the first author of the paper.

## Abstract (precis)

**Summary:** Our work found that oviductal fluid enhances sperm dynamics and survival and mitigates the negative effects of increasing temperatures on sperm in an ectotherm model.

## INTRODUCTION

Increased global surface temperatures, frequent heatwaves and extreme temperature events have been predicted by the Intergovernmental Panel on Climate Change (IPCC) for the end of the century (Allen et al., 2018; Stocker et al., 2013). Global warming is affecting biodiversity, however, there is insufficient data concerning physiological and performance impacts to extreme temperatures in natural populations (Cooper et al., 2020; Vicenzi et al., 2020). Specifically, the extent to which animal reproductive traits respond to the predicted temperature increments remains largely unexplored (Bestion et al., 2015; Dunn and Winkler, 2010; Isaac, 2009; Kingsolver et al., 2013) and reproductive sensitivity to temperature variations is better known in endotherms (Hurley et al., 2018).

By contrast, limited attention has been given to ectotherm taxa, in which reproductive processes are more directly influenced by thermal environment changes (Carey, 2014; Dubey and Shine, 2011; Paaijmans et al., 2013). In the context of climate change, both thermoregulatory behaviour and thermal physiology can be targets of selection in ectotherms (Gilbert and Miles, 2017). However, evolutionary changes in thermoregulatory capability are unlikely to keep pace with current rates of environmental change. Therefore, since the thermal environments have a pronounced impact on body temperatures of ectotherms, temperature increase is hypothesized to impact specific reproductive mechanisms, like sperm performance.

The temperature of the different pre-fertilization environments to which the sperm cells are exposed can have many fundamental effects on sperm performance and function (Fenkes et al., 2017; Sinclair et al., 2016). In fishes, increasing temperatures related to global warming affect sperm cells' metabolism (Dadras et al., 2017), beat frequency of sperm flagella and make viability decrease leading to fewer motile cells (Mansour et al., 2002). In terrestrial ectotherms such as insect groups, heatwaves compromise male fertility by reducing sperm number and viability, thus lessening its competitive ability (Gasparini et al., 2018; Sales et al., 2018). Moreover, not only average sperm parameters but also their intramale variability, which has been identified as a sperm competition strategy to maximize their fertilization success (Blengini et al., 2014; Calhim et al., 2011; Helfenstein et al., 2010), might be affected by warmer conditions.

Mechanisms in the female reproductive tract (FRT), such as sperm storage and gamete interaction, are also affected by temperature increments (Sales et al., 2018). Some mechanisms mediated by female fluids offer females the opportunity to exert post-mating female choice in several taxa (Devigili et al., 2018; Firman et al., 2017; Fitzpatrick et al., 2020). Oviductal fluid (OF) may actively enhance the motility of sperm by increasing differentially the sperm velocity of some males; this phenomenon leads to the Cryptic Female Choice (CFC) (Cardozo and Pilastro, 2018; Gasparini and Pilastro, 2011). Consequently, sperm performance in the female medium could be affected by female factors evolved as a consequence of sexual conflict (Cardozo et al., 2020; Møller et al., 2009). However, a question that has not been addressed yet is whether the role of oviductal fluid (OF) is modified due to the effects of high temperature. Rising global surface temperatures (1.1 to 6.4°C) by 2100 would increase the metabolic rates of ectotherms by 10–75%, which could negatively affect reproduction. In the light of this alarming projection (Huey et al., 2009), testing whether there is an interaction between temperature increments and OF effect on sperm performance is a mandatory first step to understanding evolutionary responses in a changing environment.

Reproductive processes may involve specific thermal requirements (e.g. preferred temperatures, a suitable thermal range and thermal stability) in ectotherms, in both males and females (Fenkes et al., 2017; López Juri et al., 2018a). Furthermore, the effects of temperature on sperm motility and velocity are related to the reproductive thermal ecology of each species (Tourmente et al., 2011a). Specifically, temperature of microenvironments plays a pivotal role in sperm performance in multiple instances, both before and after copulation (Dosemane and Bhagya, 2015). Therefore, assessing sperm performance when sperm is ejaculated, as well as under post-mating conditions, is fundamental to understand the different mechanistic bases of sperm sensitivity to temperature (Marshall, 2015).

To test the interaction between OF and sperm, effective and safe procedures to obtain both sperm and OF samples are required. In lizards, sperm collection has been addressed (López Juri et al., 2018b); however, non-lethal and non-invasive OF collection protocols are not available. In birds and fishes, some techniques have been used successfully (Cardozo and Pilastro, 2018; Cramer et al., 2014; Devigili et al., 2018; Gasparini and Pilastro, 2011). In our study, we novelly adapted them to lizards providing a thorough and safe method for OF collection to test the impact of temperature on sperm–OF interaction under a climate change scenario.

*Tropidurus spinulosus*, the spiny lava lizard, is a suitable ectotherm model for this study because reproductive individuals have specific thermal requirements (López Juri et al., 2018a). Moreover, it shows intense social reproductive interactions and male-biased operative sex ratio (López Juri, 2019), which leads to sperm competition and CFC (Pizzari and Wedell, 2013). The species distribution in temperate areas makes it an interesting model because warming would be more marked in these regions (Allen et al., 2018; Stocker et al., 2013) and because species adapted to seasonality would be more sensitive to climate change (Tang et al., 2012).

Our aim was to experimentally test the effect of increased temperature associated to an extreme global warming on sperm velocity, swimming path and percentage of motile sperm under neutral conditions at ejaculation (without OF) and to evaluate the role of female's reproductive tract fluid (with OF) on the sperm subjected to high temperature in an ectotherm model. We predict that a significant temperature increment above the preferred temperature documented for reproductive individuals will impair sperm velocity and motility whereas OF interaction will enhance the spermatic parameters.

## RESULTS

**Fig. 1.**
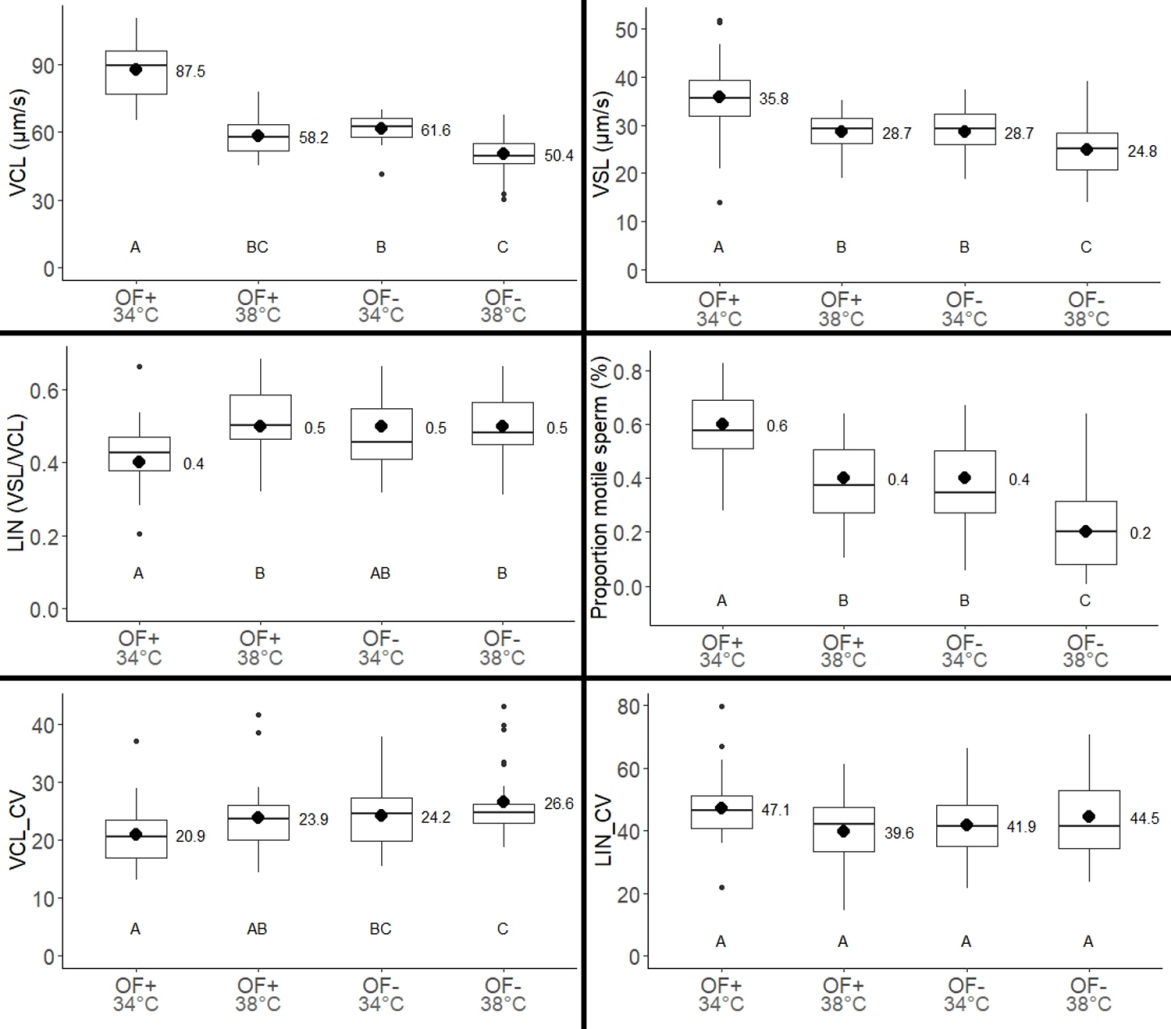
**Effects of temperature increment and oviductal fluid (OF) on sperm performance.** VSL, straight-line velocity; VCL, curvilinear velocity; LIN, linearity (LIN=VSL/VCL); motility, percentage of motile spermatozoa (%); CV, coefficients of variation. Lines within boxes correspond to the median value of the sample, the lower and upper edge of the box correspond to quartiles 1 and 3 respectively, whiskers denote the minimum and maximum values and outliers are highlighted with points. Different letters indicate significant differences according to Tukey's test (*P*<0.05). The total sample size was 27 independent replicates.

Straight line velocity (VSL; µm/s) and curvilinear velocity (VCL; µm/s) presented a similar pattern both varying among treatments (Fig. 1, Table 1). Sperm velocity was the highest at 34°C in (OF+). The increment of 4°C caused a reduction in spermatozoa velocity in both (OF−) and (OF+). However, considering the interaction, the sperm at high temperature in (OF+) was significantly faster than in (OF−). Variability in curvilinear sperm velocity (VCL) was highest when temperature increased and in (OF−).

**Table 1.**
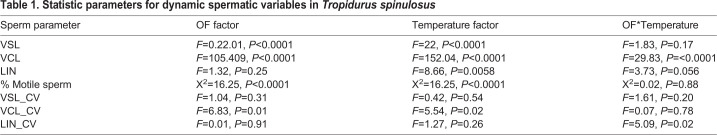
**Statistic parameters for dynamic spermatic variables in *Tropidurus spinulosus***

Under (OF−) conditions sperm linearity (LIN) was high and was not significantly modified by temperature increment. When sperm was under (OF+) conditions at 34°C spermatozoa linearity was low, however, at 38°C spermatozoa linearity increased (but with marginal significance), reaching similar values to (OF−) conditions.

The percentage of motile spermatozoa was the highest at 34°C in (OF+). Temperature increment caused a decrease in sperm motility. However, at high temperature the percentage of motile sperm in (OF+) was significantly higher than in (OF−).

## DISCUSSION

We found that a temperature increment associated to an extreme global warming affects negatively sperm velocity and percentage of motile cells. However, OF enhance sperm performance and ameliorated the negative effects of high temperature on sperm. Additionally, our method of OF extraction, based on other taxa (Cardozo and Pilastro, 2018; Møller et al., 2009) was safe and efficient, and was applied in squamates for the first time. The technique did not harm females as they did not show abnormal behaviour after the procedure (Warwick et al., 2013).

The reduction in the velocity of the spermatozoa caused by the temperature increment might impact fertilization success of vitellogenic follicles since sperm velocity is known to be a major determinant of male fertility (Beausoleil et al., 2012; Birkhead and Møller, 1998; Gomendio and Roldan, 2008). The decrease in sperm velocity might also affect the competitive ability of males; i.e. males under sperm competition risk often vary in sperm quality (Blengini et al., 2016; Cardozo et al., 2020); however, if warmer thermal environments modify sperm velocity, the ability of some males to reach the fertilization site faster might be altered.

In other squamates and mammalians, high temperatures also harm sperm dynamic, possibly through an increase in sperm metabolic rates that alters enzymes or the resistance of the lipid membrane to Reactive Oxygen Species (ROS) (Soren et al., 2018; Tourmente et al., 2011b). In ectotherm marine species, warming environments can either boost (Ho et al., 2013) or harm sperm velocity (Binet and Doyle, 2013). In internally fertilizing ectotherms, sperm production (Zeh et al., 2012) and performance within the female tract might be negatively impacted, since sperm deteriorates rapidly after being exposed to heatwaves (Sales et al., 2018).

On the other hand, although OF has already been identified as an improver of sperm motility in some fishes and birds (Rosengrave et al., 2009; Sasanami et al., 2013), in our lizard model, the OF effect on the increment of sperm velocity was notable (on average 20%) compared to that observed in other species (approximately 10% in guppies, Gasparini and Pilastro, 2011 and Cardozo and Pilastro, 2018; 10% in bovines, (Grippo et al., 1995); 12% in boars, (Coy et al., 2010); 10–17% in mouses, (Oliveira et al., 1999). The ions and protein composition naturally present in the OF may stimulate spermatozoa ATP metabolism and, thus, enhance motility (Rosengrave et al., 2009); a similar mechanism involving the pH of secreted materials in the bird's vagina might modify sperm motility (Sasanami et al., 2013).

Remarkably the negative effect of high temperature on sperm velocity seems to be less pronounced in OF than in neutral conditions. The compensation effect of OF suggests that postcopulatory mechanism mediated by FRT fluids might partially protect sperm in the oviductal tract from increasing temperatures even when the increment is above the Tset_max and near to Tmax (in *Tropidurus spinulosus* for reproductive females: Tset_max 35.34±1.40 and T_max 37.49±1.81; for reproductive males: Tset_max=35.07±2.04 and T_max 37.53±2.00; see López Juri et al., 2018a,b). As in mammalians, the compensation might be mediated by OF enzymes that inhibit sperm ROS action (Kobayashi et al., 2014) or help in restoring motility of spermatozoa under oxidative stress (Bilodeau et al., 2002). This might be important in species with prolonged mate search where sperm parameters seem to be modelled by the temperature of the habitat that males pass through (Tourmente et al., 2011a).

OF may also activate sperm through capacitation, which in reptiles has been poorly studied. Some works in crocodiles and lizards added to recent evidence that reptilian sperm may have the full range of phosphorylation-mediated cellular mechanisms and activation of mitochondria associated with capacitation, drastically increasing motility (Nixon et al., 2016). The influence of temperature on capacitation events has also been largely neglected, however the few evidence available show different impacts on motility depending on the taxa considered. In bovines, incubation gradients (20°C-40°C) affect the % of hyperactivated sperm but not sperm velocity, while in humans the opposite is true (Hammerstedt and Hay, 1980). In reptiles these processes should be studied further to properly quantify the relative contribution to motility of the capacitation mediated by OF and/or the protective effect of OF towards high temperatures.

To our knowledge velocity as a predictor of fertilization has not been tested as exclusive in squamates, so it is possible that other post-copulatory traits, such as sperm viability, are influential for fertilization success. The percentage of motile sperm is unaffected by temperature in some bird and fish species (Bonato et al., 2012), whereas high temperatures may have deleterious effects on sperm of squamates (Tourmente et al., 2011a). Our results support the latter, but also highlight the importance of OF in maintaining spermatozoa alive despite the thermal stress. Therefore, this study is the first that has ever tested the role of OF to preserve not only sperm performance but also the number of motile spermatozoa from the harmful effect of high temperature from a climate change perspective.

Sperm linearity at the preferred temperature and in OF was low. In other taxa, non-linear movement would reflect sperm cells, redirecting themselves towards a chemoattractant, like progesterone, through transitional movements (Blengini et al., 2011). Here, since the receptive females were at a late stage of vitellogenesis, progesterone level in the collected OF was probably high (Ramírez-Pinilla et al., 2009) and may have caused the observed non-linear pattern. In addition, linearity increased with temperature, which has been interpreted as a strategy to reach the follicles or storage sites earlier at the expense of lower cell viability (Mehlis and Bakker, 2014).

Variability in curvilinear sperm velocity (VCL) was highest when temperature increased and in (OF−). In other lizards, sperm variability has been found in relation to sperm competition risk (Blengini et al., 2014). In this study, the observed inter-male variability suggests differential responses of sperm performance to temperature increments, although OF apparently acts as a levelling factor. Sperm variability could be related to sperm age (Gasparini et al., 2019) or male traits e.g. body size, as occurs in *Tropidurus spinulosus* (López Juri et al., 2020). Moreover, trade-offs between colour ornamentation variability (Rossi et al., 2019) and sperm resistance to oxidative stress activated by increased temperature (Tomášek et al., 2017) might also produce variation in sperm velocity.

In this paper we focused on short-term mechanisms following ejaculation when the sperm reach the swimming environment within the female reproductive tract. However, the OF-temperature interaction might be important for longer-term postcopulatory mechanisms such as CFC mediated by female sperm storage, which is well known in lizards (Siegel et al., 2011). For example, the strong linearization of sperm trajectory that we observed might alter sperm competence to reach the oviductal crypts, thus modifying CFC and sperm precedence patterns (Magris et al., 2017). However, to test it, experiments that consider exposure to temperature for longer time should be addressed. Furthermore, submitting the animals to temperature treatment instead of testing the sole interaction between OF and sperm, may highlight thermoregulatory response mechanisms that could function as mitigation strategies. Indeed, although lizards may choose to perform certain activities even when superficial temperatures are high and body temperature in the upper preferred range (Gunderson and Leal, 2016), they may regulate their body temperature behaviourally, since most ectotherms do not have a physiological thermal-safety margin (Sunday et al., 2014). By hiding in crevices, lizards may avoid unsuitable temperatures (Cecchetto et al., 2019), thus buffering reproductive mechanisms from high temperatures.

In conclusion, our study assesses potential evolutionary responses of reproductive processes of ectotherms to global warming. Temperature increments may modify sperm dynamics and survival, possibly affecting male reproductive success. By contrast, oviductal fluid enhanced sperm performance and remarkably ameliorated the harmful effects of high temperature. These results contribute to our understanding of how global warming might affect reproductive postcopulatory mechanisms.

## MATERIALS AND METHODS

### Capture and handling

Reproductive females (*n*=15) and males (*n*=27) of *T. spinulosus* were captured in Córdoba province, Argentina (31°23′33″W, 64°35′48″S) during two consecutive breeding seasons (2017–2018) (López Juri et al., 2018a).

As reproduction in *T. spinulosus* is seasonal, we evaluated reproductive structures (by ultrasound scanning Sonosite 180 Plus) to confirm the stage of the reproductive cycle of males and females. We used receptive females with enlarged vitellogenic follicles and reproductive males with enlarged testes as they possess sperm in their deferent ducts (López Juri et al., 2018b). The individuals were isolated for one week to ensure that no recent inseminated sperm was available in the cloaca of females, and that males could replenish sperm reserves (Gasparini et al., 2019). Lizards were kept individually under light (9–17 h, UVB 5.0) and at 28°C (mean environmental temperature from capture site in their natural habitat); larvae of *Tenebrio molitor* and water were provided *ad libitum*.

This research was approved by the Governmental Environmental Agency and the Ethical Committee of the Instituto de Diversidad y Ecología Animal CONICET-UNC (protocol number: 2/2017 and 12/2019).

### Experimental design: temperature treatments in relation to IPCC projections

In brief, we collected semen and OF of individuals from wildlife. Semen of each male was tested under the concomitant effect of two factors: presence/absence of OF and two temperature levels. OF presence/absence will allow us to disentangle the effect of OF from sperm performance per se*.* In regards to temperature levels, IPCC projects climate warming within the range from 2°C to 4.5°C for the end of the century (Allen et al., 2018; Stocker et al., 2013). Moreover, in the absence of near-term mitigation to reduce emissions, the likelihood of 4°C warming being reached during the present century has increased (Fawcett et al., 2015). Consequently, we tested the following temperature treatments: (A) 34°C, which corresponds to the preferred temperature of males and receptive females during the breeding season (Tsel_females: 33.79±1.42°C; Tsel_males: 33.50±2.27°C; López Juri et al., 2018a; thermal preference is the body temperature selected in the absence of ecological constraints; see Gilbert and Miles, 2017). (B) 38°C, according to a +4°C increment in surface temperatures projected by the Representative Concentration Pathway 8.5 (RCP 8.5, Allen et al., 2018; Stocker et al., 2013). Thirty-eight degrees is close to the upper thermal range tolerated by *Tropidurus spinulosus,* however it is still a temperature they may voluntarily select to thermoregulate in a laboratory setup (López Juri et al., 2018a,b).

### OF collection

Before beginning OF collection, the cloaca was rinsed with sterile saline solution (0.9% NaCl) to eliminate any pasty urates and feces. Initially, a ventro-lateral stroking was performed to achieve relaxation of the musculature. Later the anal plate was massaged to produce distension of the cloacal musculature (Mengden et al., 1980). After that, the cloaca aperture was rubbed with a metal probe by performing circular movements. These movements favoured the relaxation of the cloacal musculature and therefore allowed us to locate the urinary papillae; the latter were gently lowered to expose the entrance to the oviduct where OF was then collected. There, 10 µl of the saline solution was injected and retrieved using a micropipette. This operation was repeated four times; a total 40 µl of saline solution was injected and approximately 25 µl of OF solution (OF+saline solution) was collected obtaining an OF dilution similar to that used in CFC studies (Cardozo and Pilastro, 2018; Gasparini et al., 2012). OF solution was examined under optical microscope to confirm the absence of recently inseminated sperm.

### Sperm collection

The lizards were electrostimulated according to (López Juri et al., 2018b) to trigger the expulsion of semen into the cloacal ampulla. We added 10 µl of saline solution to the expelled semen: this mixture was immediately aspired with a micropipette and then rinsed with into 10 µl of saline solution. This procedure was repeated until all samples were completed to a fixed volume of 20 µl of semen solution (semen+saline solution). A mean sperm number of 2.1±1.8×10^6^ spermatozoa was obtained. Because the number of captured receptive females was nearly half of reproductive males, the OF solution from each female was usually tested with sperm samples of two males.

### Animal welfare

After semen/OF collection, lizards were checked once a week for normal alertness, calm chemical sample of surrounding, unhurried locomotion, relaxed feeding and breathing (Warwick et al., 2013). After that, the specimens were released at the capture sites (GPS coordinates).

### Experimental protocol

To simulate sperm ejaculation and post-mating conditions, sperm velocity was measured in two different solutions (Fig. S1): (A) OF absence treatment (OF−): consisting of 8 µl (40% volume) of Ham's F-10 culture medium (Ham's F-10. Gibco, New York, USA), supplemented with 1% bovine serum albumin, and 12 µl (60% volume) of saline solution; (B) OF presence treatment (OF+): consisting of 8 µl (40% volume) of similarly supplemented Ham's F-10 culture medium and 12 µl (60% volume) of OF solution; this proportions were adjusted according to (Gasparini and Pilastro, 2011). Semen solution (2 µl) was added to the A and B solutions, in random order. The total volume (22 µl) of each solution was then divided and used for both incubation temperature treatments: 34°C and 38°C. Thus, the sample series was composed of four treatments: (1) OF+ 34°C, (2) OF+ 38°C, (3) OF− 34°C, and (4) OF− 38°C. Samples were incubated in thermal baths (Thermo Scientific Precision™) for 15 min, which is sufficient to observe relevant effects on motility (Awda et al., 2009; Williams and Ford, 2005), and then processed in a randomized fashion. The total number of series was 27.

### Sperm parameters

Dynamic parameters were measured using a video microscopy system (Eclipse 50i Nikon phase contrast; camera Nikon Digital Sight DS-Fi2). Sperm tracks were followed for 3 s (mean±s.d.=21±3 cells/sample) and transformed to a matrix of Cartesian coordinates using ImageJ version 1.43u (NIH) and its plug-in MtrackJ v. 1.1.0 (Meijering et al., 2012). Sperm dynamic parameters were calculated using Spermtrack v. 4.2 (Universidad Nacional de Cordoba, Argentina): straight-line velocity (VSL; µm/s), curvilinear velocity (VCL; µm/s), and swimming path estimated as linearity (LIN=VSL/VCL). The percentage of motile spermatozoa was estimated over approximately 50 sperm cells per analysis with random changes of the microscope fields. Variability of the motility parameters (VSL, VCL and LIN) was evaluated by calculating Coefficients of Variation (CV), which is a useful statistics to evaluate sperm variability associated with motility (Blengini et al., 2014; Immler et al., 2008).

### Statistical analysis

We fitted a linear mixed effect models with ‘female ID’, ‘male ID’ and ‘year’ as random effects and ‘temperature’ and ‘OF treatment’ and their interaction as fixed factors (package lme4, Bates et al., 2015). Percentage of motile spermatozoa was tested with a generalized linear model with a binomial link function and the same random and fixed effects as the linear models. Since ‘year’ was always non-significant, we removed it from all models. Normality and homoscedasticity were checked by inspecting residuals distribution. Shapiro–Wilks test was also performed when checking linear models. A *posteriori* Tukey test was performed to determine differences among treatments. Statistical analyses were performed in R version 3.6.1 (R Core Team, 2019).

## Supplementary Material

Supplementary information
