# Supplementary material for: Oviductal fluid counterbalances the negative effect of high temperature on sperm in an ectotherm model
Source: Biol Open. 2021 Apr 15;10(4):bio058593. doi: 10.1242/bio.058593 (PMC8061905; doi:10.1242/bio.058593)
Supplement: Supplementary information [file biolopen-10-058593-s1.pdf]

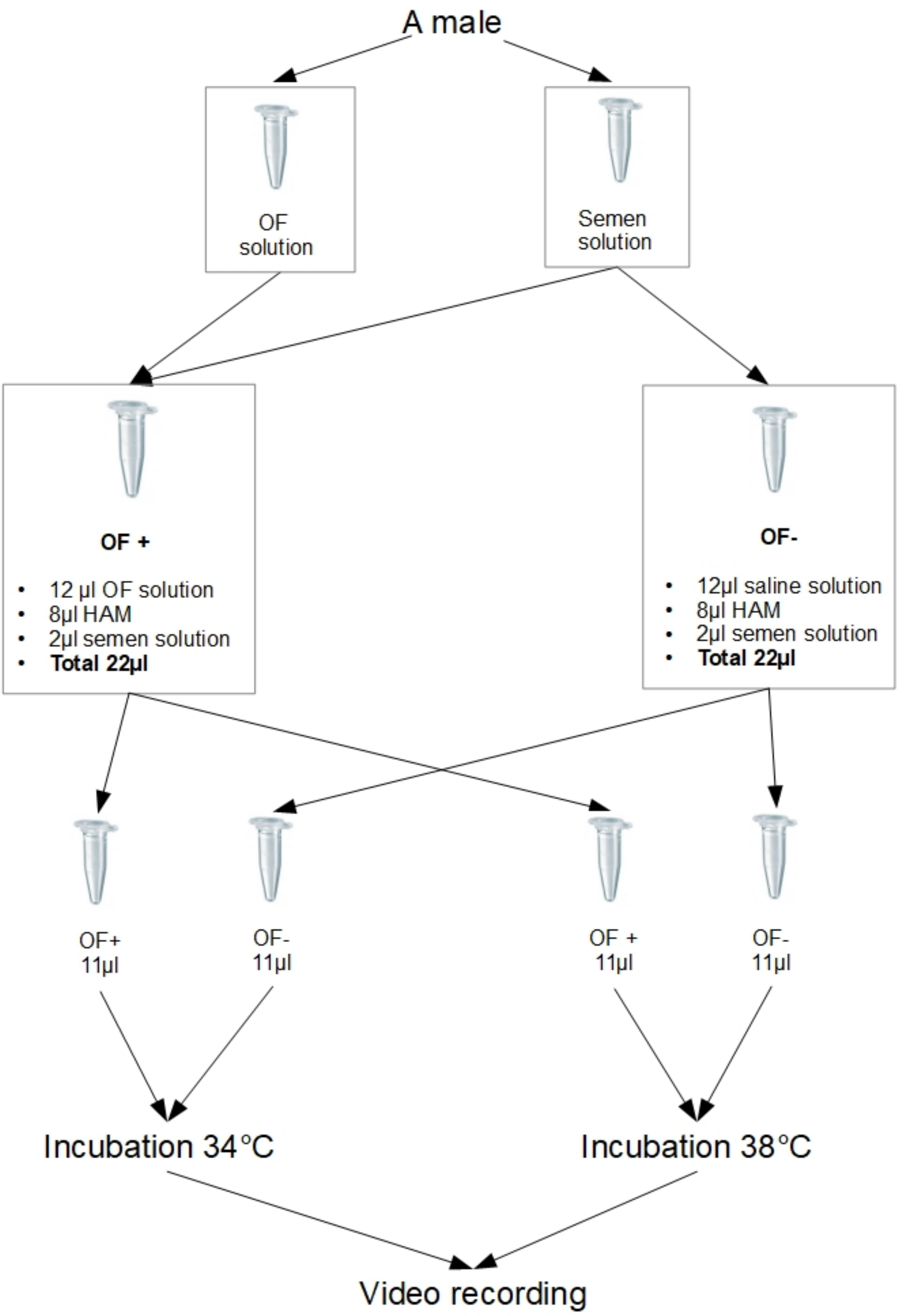

Fig. S1. Schematic representation of the experimental protocol to assess the influence of oviductal fluid (OF) and temperature on sperm dynamics and viability in Tropidurus spinulosus

Biology Open • Supplementary information
